# Outcomes of aortic and mitral valve replacement with Dafodil™ pericardial bioprosthesis over 5-years: Dafodil™-1 trial

**DOI:** 10.3389/fcvm.2025.1672315

**Published:** 2026-01-09

**Authors:** Channabasavaraj Shivalingaiah Hiremath, Anil Jain, Anurag Garg, Atul Maslekar, Nirmal Gupta, Binay Krishna Sarkar, Seetharama Bhat, Manish Porwal, Zile Singh Meharwal, Yugal Kishore Mishra, Prashant Vaijyanath, Vijay Grover, Shiv Kumar Chaudhary, Subash. S. Rajput, Rajan Sethuratnam, Naman Shastri, Chandrasekar Padmanabhan, Nityanand Tukaram Thakur, Chittaranjan S. J., Dhaval Naik, Vijay Kumar Gupta, Anand Sancheti

**Affiliations:** 1 Sri Madhusudan Sai Institute of Medical Sciences and Research, Sri Sathya Sai Sarala Memorial Hospital, Sathya Sai Grama, Muddenahalli, Chikkaballapur, India; 2 Department of Cardiovascular and Thoracic Surgery, EPIC Hospital, Ahmedabad, India; 3 Department of Cardiothoracic Surgery, Dr. D.Y. Patil Medical College & Hospital & Research Centre, Pune, India; 4 Department of Cardiothoracic and Vascular Surgery, Narayana Multispeciality Hospital, Ahmedabad, India; 5 Department of Cardiovascular and Thoracic Surgery, Sanjay Gandhi Postgraduate Institute of Medical Sciences, Lucknow, India; 6 Department of Cardiothoracic and Vascular Surgery, Nil Ratan Sircar Medical College & Hospital, Kolkata, India; 7 Department of Cardiothoracic and Vascular Surgery, Sri Jayadeva Institute of Cardiovascular Sciences & Research, Bangalore, India; 8 Department of Cardiothoracic Surgery, Convenient Hospitals Limited (CHL), Indore, India; 9 Department of Cardiothoracic and Vascular Surgery, Fortis Escorts Heart Institute, New Delhi, India; 10 Department of Cardiothoracic and Vascular Surgery, Manipal Hospital, Delhi, India; 11 Department of Cardiothoracic Surgery, Kovai Medical College and Hospital, Coimbatore, India; 12 Department of Cardiothoracic and Vascular Surgery, Dr. Ram Manohar Lohia Hospital, New Delhi, India; 13 Department of Cardiothoracic and Vascular Surgery, Ram Manohar Lohia Institute of Medical Sciences, Lucknow, India; 14 Department of Cardiac Surgery, The Madras Medical Mission Hospital, Chennai, India; 15 Division of Cardiothoracic Surgery, G Kuppuswamy Naidu Memorial Hospital, Coimbatore, India; 16 Department of Cardiothoracic Surgery, B.J.Govt. Medical College & Sassoon General Hospitals, Pune, India; 17 Cardiac & Heart Transplant Surgery, Care Institute of Medical Sciences (CIMS), Ahmedabad, India; 18 Department of Cardiothoracic & Vascular Surgery, Apollo, Delhi, India; 19 Cardiothoracic and Vascular Surgery, New Era Hospital & Research Institute, Nagpur, India

**Keywords:** aortic valve, heart valve prosthesis implantation, mitral valve, pericardial bioprosthesis, quality of life, valvular heart disease

## Abstract

**Objectives:**

The durability and performance of bioprosthetic valves are pivotal in managing valvular heart disease (VHD). This 5-year follow-up study evaluated the long-term clinical safety, haemodynamic performance, and quality-of-life (QoL) outcomes of the Dafodil™ pericardial bioprosthesis in patients undergoing aortic or mitral valve replacement (AVR/MVR).

**Methods:**

In this prospective, multicenter, first-in-human trial conducted across 19 Indian centers, 136 patients with advanced VHD (67 AVR, 69 MVR) were enrolled. Key endpoints were evaluated over 5 years.

**Results:**

The mean age of the patients in the AVR and MVR cohorts was 60.2 ± 8.3 years and 49.7 ± 14.4 years, respectively. A total of 124 patients (61 AVR, 63 MVR) completed the 5-year follow-up. In the AVR cohort, mean pressure gradients decreased from 51.2 ± 24.1 mmHg at baseline to 11.3 ± 5.3 mmHg at five years, while effective orifice area (EOA) increased from 0.88 ± 0.56 cm^2^ to 1.72 ± 0.52 cm^2^ (*p* < 0.001). In the MVR group, the indexed EOA improved from 0.68 ± 0.4 cm^2^/m^2^ to 1.28 ± 0.39 cm^2^/m^2^ (*p* < 0.001). Both cohorts exhibited significant improvements in New York Heart Association (NYHA) class and SF-12 QoL scores. There were no cases of haemolysis, structural valve deterioration (SVD), or myocardial infarction. At five years, the incidence of all cause of mortality was 9.8% in the AVR group and 22.2% in the MVR group. The major adverse cardiac event-free survival rates were 88.0% (AVR) and 76.7% (MVR).

**Conclusions:**

Over five years, the bioprosthesis demonstrated sustained haemodynamic performance, clinical safety, and QoL. The absence of SVD and low rates of adverse events encourage its use in young patients requiring surgical valve replacement. Furthermore, a long-term follow-up is warranted to evaluate its durability.

## 1 Introduction

Valvular heart disease (VHD) affects an estimated 40.5 million people worldwide, with aortic and mitral valve pathogenesis significantly contributing to global cardiovascular morbidity (1). 40% of people with rheumatic heart disease (RHD) live in India. A total of 13.2 million of the estimated 33 million individuals with RHD reside in India. Similarly, more than one-third of the 347,000 RHD-related deaths that occurred globally in 2015 are thought to have happened in India (2). In Asia, rheumatic aortic stenosis is prevalent, with a rate of 4.54 cases per 1,000 cases in India (1). The primary interventions for surgical treatment of aortic valve disease are transcatheter aortic valve replacement (TAVR) and surgical aortic valve replacement (SAVR). SAVR remains the standard of care for patients with aortic valve disease (3, 4). SAVR is also the preferred option in patients requiring concomitant cardiac procedures (5).

Contemporary guidelines from the American College of Cardiology (ACC)/American Heart Association (AHA) (2020) and the European Society of Cardiology (ESC)/European Association for Cardio-Thoracic Surgery (EACTS) (2025) differ slightly in their thresholds but consistently support SAVR for younger patients at low surgical risk (6). Both guidelines support the use of bioprosthetic valves for patients aged <70 years, reaffirming the relevance of SAVR in this age group (7). Considering that a notable number of patients were under 50 years of age, it is important to note that there is evidence supporting the use of bioprosthetic SAVR in chosen younger patients, particularly when anticoagulant avoidance and lifestyle considerations are prioritized. Hence, in this age group it is justifiable to evaluate the long-term outcomes of the Dafodil™ pericardial bioprosthesis (Meril Life Sciences, India), which is an indigenously developed tri-leaflet stented bovine pericardial valve designed for supra-annular placement in the aortic position and intra-annular implantation in the mitral position, offering enhanced coaptation and anti-calcification properties. It comes in a variety of sizes suitable for both aortic and mitral valve positions, ensuring a precise anatomical fit. Despite mitral valve repair being unfeasible, MVR remains necessary, especially in rheumatic mitral disease, which continues to be common. MVR is often recommended in patients with severely debilitating mitral stenosis or regurgitation, particularly those with valves that are calcified or heavily deformed. However, the decision between using mechanical and bioprosthetic valves in this age group remains complex. Although both mechanical and bioprosthetic valves offer unparalleled durability, the clinical use of mechanical valves are often limited due to their need for lifelong anticoagulation. Although bioprosthetic valves are vulnerable to SVD over time, significant technological advances have now enabled them to increase their use (8–12).

The majority of bioprosthetic valves currently being utilized are imported, expensive, and not specifically tailored to the anatomical and clinical profiles of patients in India. There is a pressing need for bioprostheses developed in the country that are cost-effective, with proven long-term safety, haemodynamic performance, and resistance to SVD in both aortic and mitral positions.

Early results from the Dafodil™-1 trial demonstrated good safety and haemodynamic outcomes at 1 year in 60 patients (12). At 3 years, data from 136 patients showed sustained performance, low thrombosis rates, and no SVD (13). This study reports the 5-year results, evaluating the long-term clinical safety, haemodynamics, and quality of life (QoL) of patients who underwent SAVR or mitral valve replacement (MVR) using the Dafodil™ bioprosthesis.

## 2 Materials and methods

### 2.1 Study design

The Dafodil™-1 trial (Clinical Trials Registry, India number: CTRI/2017/07/009008) was a first-in-human, prospective, multicenter study initially enrolled 60 patients at 7 centres around India as early phase of the study. The trial was expanded to 19 centers between July 2017 and July 2019, during which a total of 193 participants were screened. Of these, 136 patients meeting the eligibility criteria were ultimately enrolled for the final analysis. As per the recommendation and approval provided by all the participating author, we believe that, this expanded patient cohort significantly strengthens this study's statistical power and generizability of the 5-year data. The institutional ethics committee of each participating site approved the protocol. Written informed consent was obtained from all patients. Eligible patients were adults (≥18 years) with symptomatic severe aortic or mitral valve disease and a Society of Thoracic Surgeons Predicted Risk of Mortality (STS-PROM) of <4%.

The surgical technique and anti-thromboembolic therapy was performed at the surgeon's discretion. Valve sizes used in the study were 19 mm, 21 mm, 23 mm, and 25 mm in the aortic position and 25 mm, 27 mm, 29 mm, and 31 mm in the mitral position. Patients were evaluated preoperatively, post-procedure, at 1 month, 6 months, and annually for up to 5 years. A detailed methodology, including the study design, eligibility criteria, surgical technique, echocardiographic assessment protocols, definitions of clinical endpoints, and outcomes up to 3 years, has been previously reported (12, 13). A summary of the study endpoints evaluated in this trial is provided in Supplementary Table S1. Clinical safety outcomes were measured through primary endpoints, which included major adverse cardiovascular events (MACE), all-cause mortality, myocardial infarction, stroke, valve thrombosis, and valve-related reoperation. The secondary endpoints included haemodynamic performance (effective orifice area, mean pressure gradients), New York Heart Association (NYHA) functional class, and health-related quality of life (QoL) measured by SF-12 scores.

### 2.2 Statistical analysis

Data analyses were performed using the Statistical Package for the Social Sciences (SPSS; version 22.0; IBM, Armonk, New York, USA). The study sample size was determined using a non-probability sampling approach, with a target enrolment of at least 120 patients (60 in the AVR group and 60 in the MVR group), considering potential dropouts. An intention-to-treat approach was adopted. Continuous variables are summarised as mean ± standard deviation, while categorical variables are reported as counts and percentages. Comparisons across time points were performed using paired *t*-tests. At the five-year time point, clinical events are reported as cumulative percentages (%) rather than per 100 patient-years, as all patients included in the analysis completed a uniform five-year follow-up. The standard reporting in comparable surgical valve studies with fixed duration follow-up is aligned with this approach. Kaplan–Meier curves were constructed for survival analyses. Statistical significance was defined as a two-sided *p*-value <0.05.

## 3 Results

**Figure 1:**
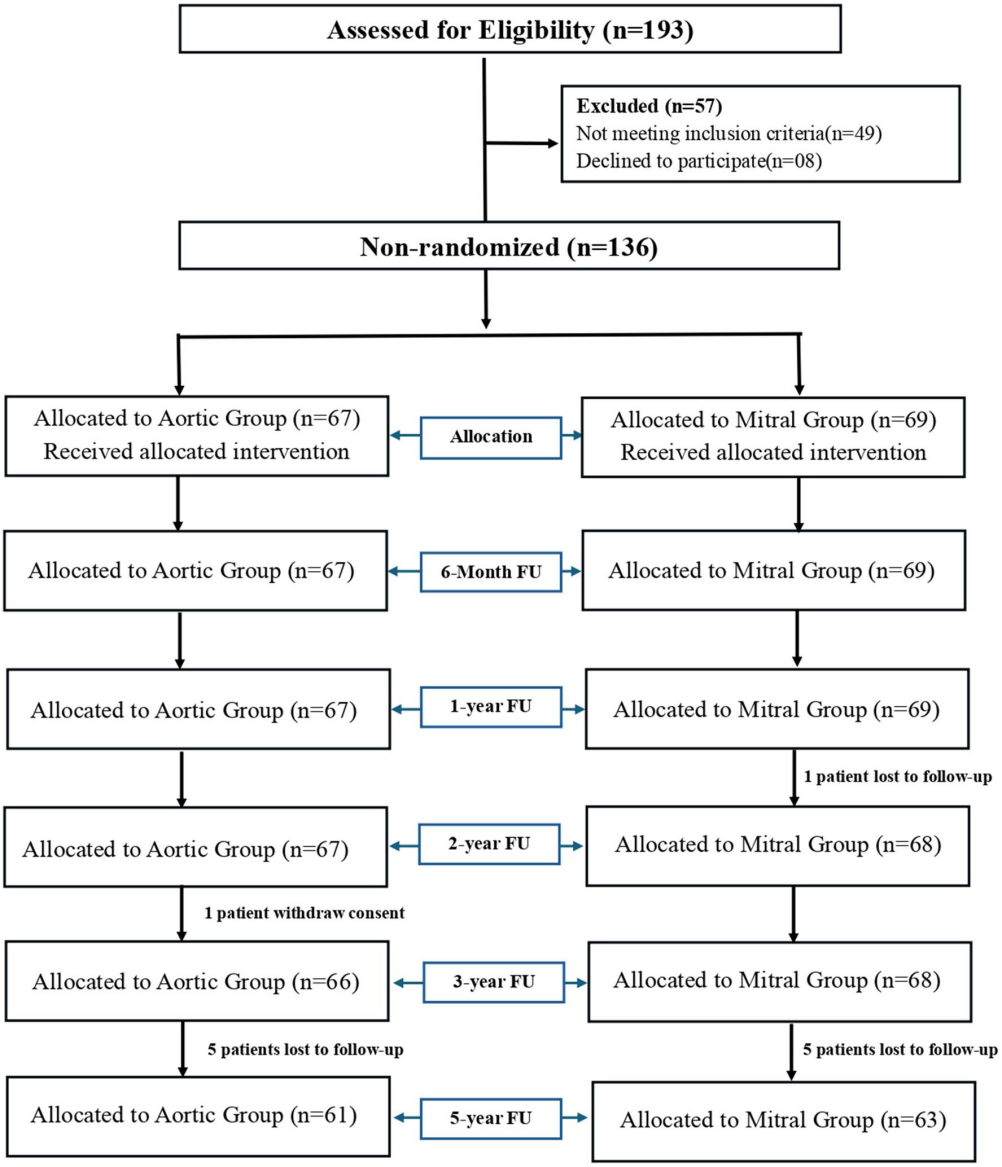
CONSORT flow diagram.

Clinical and procedural outcomes for this cohort have been previously reported over 3 years. This study provides extended follow-up findings up to 5 years, including consolidated baseline characteristics, safety, haemodynamic performance, and QoL outcomes. Of the 193 patients screened, 136 were enrolled in the study (AVR, 67; MVR, 69). At the 5-year follow-up, AVR group had five patients (7.46%) lost to follow-up and one (1.49%) patient who withdrew consent, while the MVR group had six patients (8.69%) lost to follow-up. In total, 124 patients (91.2%) (AVR, 61; MVR, 63) completed the 5-year follow-up period (Figure 1).

### 3.1 Baseline characteristics

Patients in the AVR group had a mean age at baseline of 60.2 ± 8.3 years, and 65.7% (*n* = 44) of the participants were male. The mean STS-PROM score was 1.3% ± 0.7%, indicating low surgical risk. In contrast, the MVR group had a mean age at baseline of 49.7 ± 14.4 years, with 40.6% (*n* = 28) of the patients being male. The mean STS-PROM score was 1.7% ± 1.3%. The baseline clinical characteristics, including comorbidities and cardiac history, are provided in Supplementary Table S2.

### 3.2 Safety outcomes

#### 3.2.1 AVR group

At discharge, the major adverse cardiovascular event (MACE) rate was 1.5%, with no cardiovascular mortality. At the 5-year follow-up, the MACE rate was 13.11%, which included stroke (6.56%) and all-cause mortality (9.84%). No cases of SVD, myocardial infarction (MI), major bleeding, valve thrombosis, prosthetic valve endocarditis, haemolysis, or valve-related reoperation were reported during the 5-year follow-up. However, one patient (1.64%) developed a major paravalvular leak by year 5, and three (4.92%) required permanent pacemaker implantation for conduction disturbances (Table 1).

**Table 1:** Clinical outcomes.

Events, *n* (%)	Early outcomes				Late outcomes					
	Post procedure (Discharge) (*n* = 136)		One-month follow up (*n* = 136)		One-year follow up (*n* = 136)		Four-years follow up (*n* = 130)		Five-years follow up (*n* = 124)	
	Aortic (*n* = 67)	Mitral (*n* = 69)	Aortic (*n* = 67)	Mitral (*n* = 69)	Aortic (*n* = 67)	Mitral (*n* = 69)	Aortic (*n* = 64)	Mitral (*n* = 66)	Aortic (*n* = 61)	Mitral (*n* = 63)
All-cause mortality	1 (1.5)	3^a^^,^^b^ (4.3)	2 (3.0)	4 (5.8)	2 (3.0)	9 (13.0)	6^c^^,^^f^ (9.38)	14^d^^,^^e^ (21.21)	6 (9.84)	14 (22.22)
Cardiovascular death	0	2 (3.0)	1 (1.5)	3 (4.3)	1 (1.5)	6 (8.7)	2 (3.13)	11 (16.67)	2 (3.28)	11 (17.46)
Myocardial infarction	0	0	0	0	0	0	0	0	0	0
Stroke	0	0	1 (1.5)	0	3^f^ (4.5)	2 (2.9)	4^c^ (6.25)	2 (3.03)	4 (6.56)	2 (3.17)
MACE	1 (1.5)	3 (4.3)	3 (4.5)	4 (5.8)	5 (7.5)	11 (15.9)	8 (12.5)	16 (24.24)	8 (13.11)	16 (25.4)
All Bleeding (major & minor)	0	1^d^ (1.4)	0	1 (1.4)	0	1 (1.4)	0	1 (1.52)	0	1 (1.59)
Valve thrombosis	0	1^a^ (1.4)	0	1 (1.4)	0	1 (1.4)	0	1 (1.52)	0	1 (1.59)
Structural valve deterioration	0	0	0	0	0	0	0	0	0	0
Repeat hospitalization	0	0	4 (6.0)	5 (7.2)	8 (11.9)	9 (13.0)	13 (20.31)	15 (22.73)	14 (22.95)	16 (25.40)
Conduction disturbances and arrhythmias/Permanent pacemaker implantation	1 (1.5)	0	2 (3.0)	0	2 (3.0)	0	3 (4.69)	0	3 (4.92)	2 (3.17)
Prosthetic valve endocarditis	0	0	0	0	0	0	0	1 (1.52)	0	1 (1.59)
Major paravalvular leak	0	0	0	0	0	0	0	2^d^ (3.03)	1 (1.64)	2 (3.17)
Explant	0	1^a^ (1.4)	0	1 (1.4)	0	1 (1.4)	0	3^d^^,^^e^ (4.55)	0	3 (4.76)
Haemolysis	0	0	0	0	0	0	0	0	0	0
Valve-related re-operation	0	1^a^ (1.4)	0	1 (1.4)	0	1 (1.4)	0	3^d^^,^^e^ (4.55)	0	3 (4.76)

MACE, major adverse cardiovascular event.

MACE is defined as a composite of all-cause mortality, MI, and stroke.

a One patient had valve thrombosis postoperatively and had to re-operate eventually died.

b One patient had excessive bleeding postoperatively and succumbed to death.

c One patient suffered from CV stroke at 3 years FU and eventually succumbed to death at 4 years FU.

d One patient suffered from severe PVL, explant, redo MVR and eventually succumbed to death at 4 years FU.

e One patient died due to Multi-Organ Dysfunction who underwent redo surgery at 3-year follow-up.

f One patient suffered from brain stroke at 1 year and eventually died at 2-year follow-up.

#### 3.2.2 MVR group

At discharge, MACE and cardiovascular mortality rates were 4.3% and 3.0%, respectively. At the 5-year follow-up, the MACE rate was 25.4%, which included a stroke rate of 3.17% and an all-cause mortality rate of 22.22%. No SVD, myocardial infarction, or haemolysis was observed. Still, several other late complications did occur: major paravalvular leak in 3.17%, explant in 4.76%, valve-related reoperation in 4.76%, prosthetic valve endocarditis in 1.59%, permanent pacemaker implantation for conduction disturbances in 3.17%, bleeding (major or minor) in 1.59%, and valve thrombosis in 1.59% (Table 1). The result of linearized rate for all complication with Objective performance criteria (OPC) (14) for Aortic and Mitral groups is shown in Table 2.

**Table 2:** Valve-related adverse events.

Adverse event	Aortic		Mitral	
	Linearized late event rate	Upper boundary OPC	Linearized late event rate	Upper boundary OPC
Major bleeding	0	1.2	0.33	1.4
Major paravalvular leak	0.31	0.6	0.66	0.4
Prosthetic valve endocarditis	0	1	0.33	0.8
Valve thrombosis	0	0.08	0.33	0.06

OPC, objective performance criteria.

**Figure 2:**
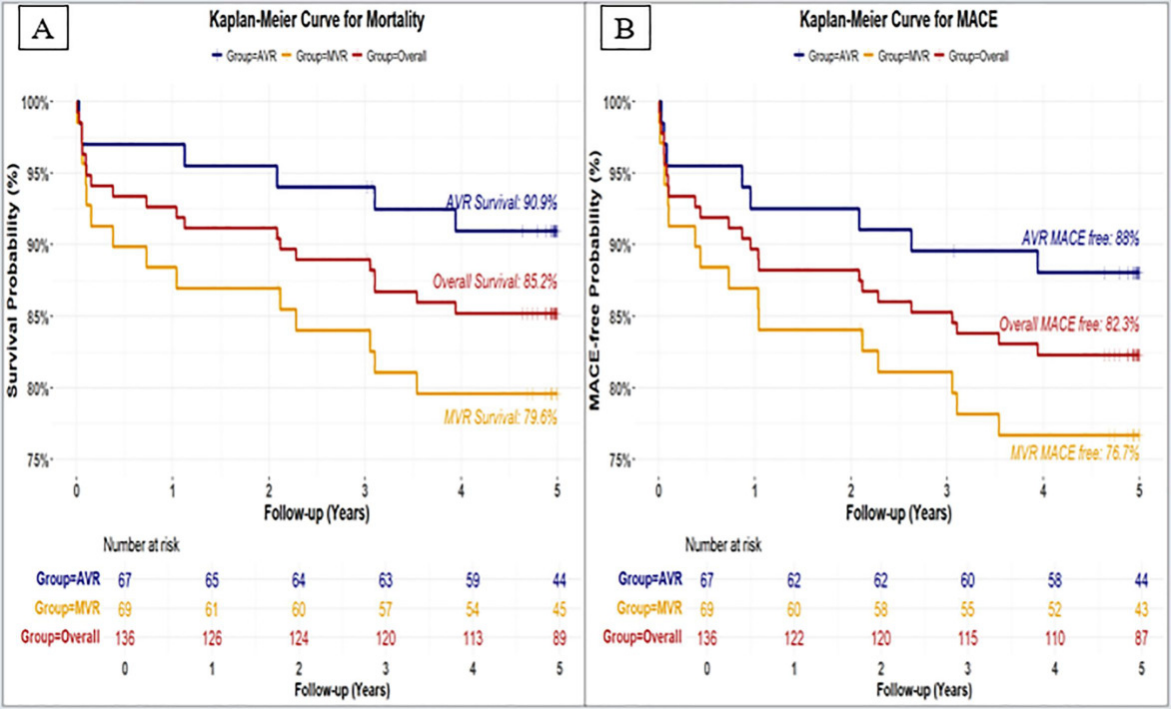
Kaplan–Meier estimates of overall survival and MACE-free survival in patients undergoing aortic valve replacement (AVR) or mitral valve replacement (MVR). **(A)** Kaplan–Meier estimates of overall survival in AVR group and MVR group, **(B)** Kaplan–Meier estimates of MACE-free survival in AVR group and MVR group.

MACE-free and overall survival rates at 5 years were higher in the AVR group than in the MVR group. The survival rates was 90.9% in the AVR group and 79.6% in the MVR group with 85.2% overall survival rates (Figure 2A), whereas MACE-free survival rates were 88.0% and 76.7% in the AVR and MVR groups with 82.3% overall MACE-free survival rates, respectively (Figure 2B). Several deaths were preceded by adverse clinical events, such as paravalvular leak, or multi-organ dysfunction, according to a review of clinical records. Notably, three patients in the MVR group underwent valve explantation due to valve-related complications, namely significant paravalvular leak, valve thrombosis, and prosthetic dysfunction requiring redo mitral valve replacement. The mortality of all three cases highlighted the clinical severity in these cases.

### 3.3 Haemodynamic outcomes

#### 3.3.1 AVR group

Echocardiographic data for the AVR cohort are presented in Table 3. Significant haemodynamic improvements were reported at 5 years from the baseline. The mean effective orifice area (EOA) significantly increased from 0.88 ± 0.56 cm^2^ at baseline to 1.72 ± 0.52 cm^2^ at 5 years (*p* < 0.001). The mean pressure gradient significantly decreased from 51.16 ± 24.07 mmHg at baseline to 11.25 ± 5.28 mmHg (*p* < 0.001) at 5-year. Findings related to early post-procedural aortic regurgitation (AR) have been detailed in earlier publications. No patient exhibited severe aortic regurgitation (AR) following valve implantation. At 5 years, none of the patients reported moderate or severe AR (Table 3).

**Table 3:** Echocardiographic analysis.

Metrics	Early outcomes			Late outcome		
	Baseline	Discharge	1 Month	1 Year	4 Years	5 Years
Aortic						
Peak pressure gradient (mmHg)	80.41 ± 33.99	25.53 ± 14.28	23.14 ± 6.65	24.59 ± 9.72	20.78 ± 9.24	21.75 ± 9.91
Mean pressure gradient (mmHg)	51.16 ± 24.07	13.87 ± 7.57	12.44 ± 3.82	13.83 ± 6.29	10.49 ± 4.76	11.25 ± 5.28
EOA (cm^2^)	0.88 ± 0.56	1.53 ± 0.53	1.57 ± 0.40	1.67 ± 0.39	1.58 ± 0.46	1.72 ± 0.52
EOAI (cm^2^/m^2^)	0.54 ± 0.34	0.94 ± 0.33	0.96 ± 0.24	1.04 ± 0.25	0.97 ± 0.33	1.04 ± 0.4
Aortic Regurgitation Grade	*n* = 50	*n* = 54	*n* = 52	*n* = 46	*n* = 42	*n* = 46
Nil	18 (36.00)	45 (83.33)	45 (86.54)	38 (82.61)	35 (83.33)	34 (73.91)
Trace/Trivial	1 (2.00)	4 (7.41)	5 (9.62)	6 (13.04)	3 (7.14)	9 (19.57)
Mild	9 (18.00)	3 (5.56)	0 (0.00)	1 (2.17)	3 (7.14)	3 (6.52)
Moderate	19 (38.00)	2 (3.70)	2 (3.85)	1 (2.17)	1 (2.38)	0 (0.00)
Severe	3 (6.00)	0 (0.00)	0 (0.00)	0 (0.00)	0 (0.00)	0 (0.00)
Mitral						
Peak pressure gradient (mmHg)	16.59 ± 8.27	10.17 ± 4.18	9.17 ± 3.81	10.49 ± 5.89	10.57 ± 4.44	10.59 ± 4.6
Mean pressure gradient (mmHg)	8.83 ± 4.95	3.92 ± 1.62	3.65 ± 1.76	4.38 ± 2.82	4.36 ± 1.87	4.37 ± 2.76
EOA (cm^2^)	1.07 ± 0.59	1.83 ± 0.52	1.87 ± 0.51	1.68 ± 0.70	1.55 ± 0.68	1.79 ± 0.57
EOAI (cm^2^/m^2^)	0.68 ± 0.44	1.25 ± 0.42	1.24 ± 0.36	1.10 ± 0.46	0.99 ± 0.4	1.24 ± 0.37
Mitral Regurgitation grade	*n* = 44	*n* = 46	*n* = 43	*n* = 35	*n* = 32	*n* = 35
Nil	5 (11.36)	37 (80.43)	33 (76.74)	22 (62.86)	21 (65.63)	25 (71.43)
Trace/Trivial	10 (22.73)	4 (8.70)	5 (11.63)	8 (22.86)	5 (15.63)	4 (11.43)
Mild	13 (29.55)	5 (10.87)	4 (9.30)	3 (8.57)	6 (18.75)	6 (17.14)
Moderate	6 (13.64)	0 (0.00)	1 (2.33)	2 (5.71)	0 (0.00)	0 (0.00)
Severe	10 (22.73)	0 (0.00)	0 (0.00)	0 (0.00)	0 (0.00)	0 (0.00)

EOA, effective orifice area; EOAI, effective orifice area index.

#### 3.3.2 MVR group

Haemodynamic performance of the mitral prosthesis remained favourable for 5 years. Sustained valve performance was observed at 4 years (1.55 ± 0.68 cm^2^) and at 5 years (1.79 ± 0.57 cm^2^). The mean pressure gradient declined from 8.83 ± 4.95 mmHg at baseline to 4.37 ± 2.76 mmHg at 5 years. The mean EOA increased significantly from 1.07 ± 0.59 cm^2^ at baseline to 1.79 ± 0.57 cm^2^ at 5 years (*p* < 0.001), indicating sustained leaflet mobility and valve competence over time. None of the patients demonstrated moderate or severe MR at any follow-up time point (Table 3).

### 3.4 New York heart association functional status and QoL

#### 3.4.1 AVR group

**Figure 3:**
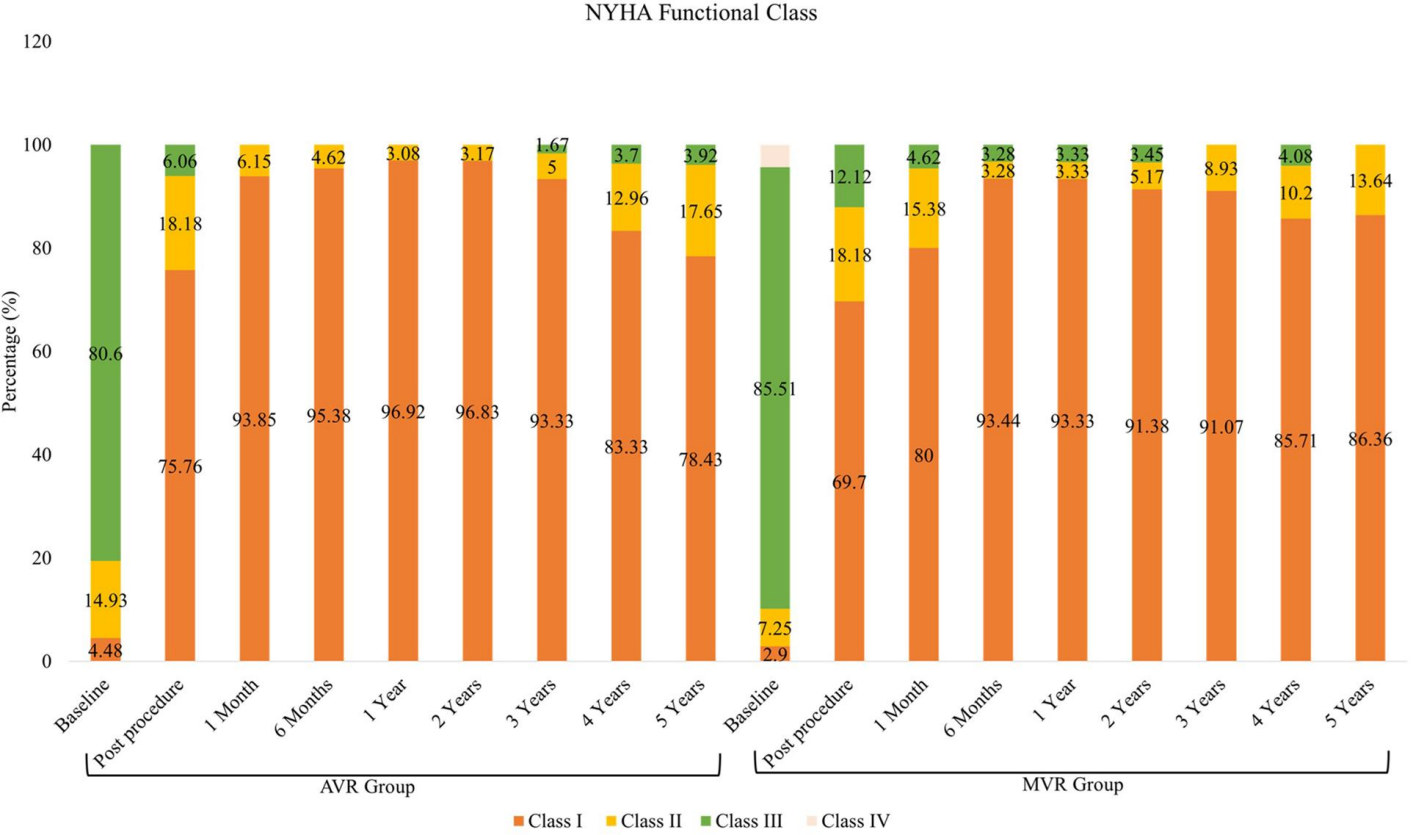
NYHA functional class: AVR and MVR groups.

At enrolment, only 4.5% of the patients were classified as New York Heart Association (NYHA) functional class I. Postoperative functional status improved markedly with 78.4% at 5 years (Figure 3). The physical component summary (PCS) score increased from 33.8 ± 7.4 at baseline to 46.38 ± 7.44 at 5 years (*p* < 0.001), while the mental component summary (MCS) score improved from 43.4 ± 9.6 to 53.1 ± 9.2 (*p* < 0.001) (Supplementary Table S3).

#### 3.4.2 MVR group

NYHA functional class I was present in only 2.9% of the patients at baseline. Following surgery, functional class I was observed in 86.36% at 5 years. Notably, no patients were classified as NYHA class III or IV at 5 years (Figure 3). The PCS score increased from 32.05 ± 6.36 at baseline to 44.78 ± 7.91 at 5 years (*p* < 0.001), and the MCS score rose from 43.49 ± 10.15 to 51.82 ± 8.63 (*p* < 0.001) (Supplementary Table S3).

### 3.5 Patients with small aortic annulus (AVR)

In the 19 mm group, the mean pressure gradient decreased from 54.3 ± 25.1 to 12.1 ± 4.75 mmHg at 5 years (*p* < 0.01), and in the 21 mm group from 56.4 ± 19.7 to 11.82 ± 6.4 mmHg (*p* < 0.001). EOA increased from 0.8 ± 0.3 to 1.46 ± 0.36 cm^2^ in the 19 mm group (*p* < 0.001) and to 1.72 ± 0.46 cm^2^ in the 21 mm group (Supplementary Table S4).

## 4 Discussion

**Figure 4:**
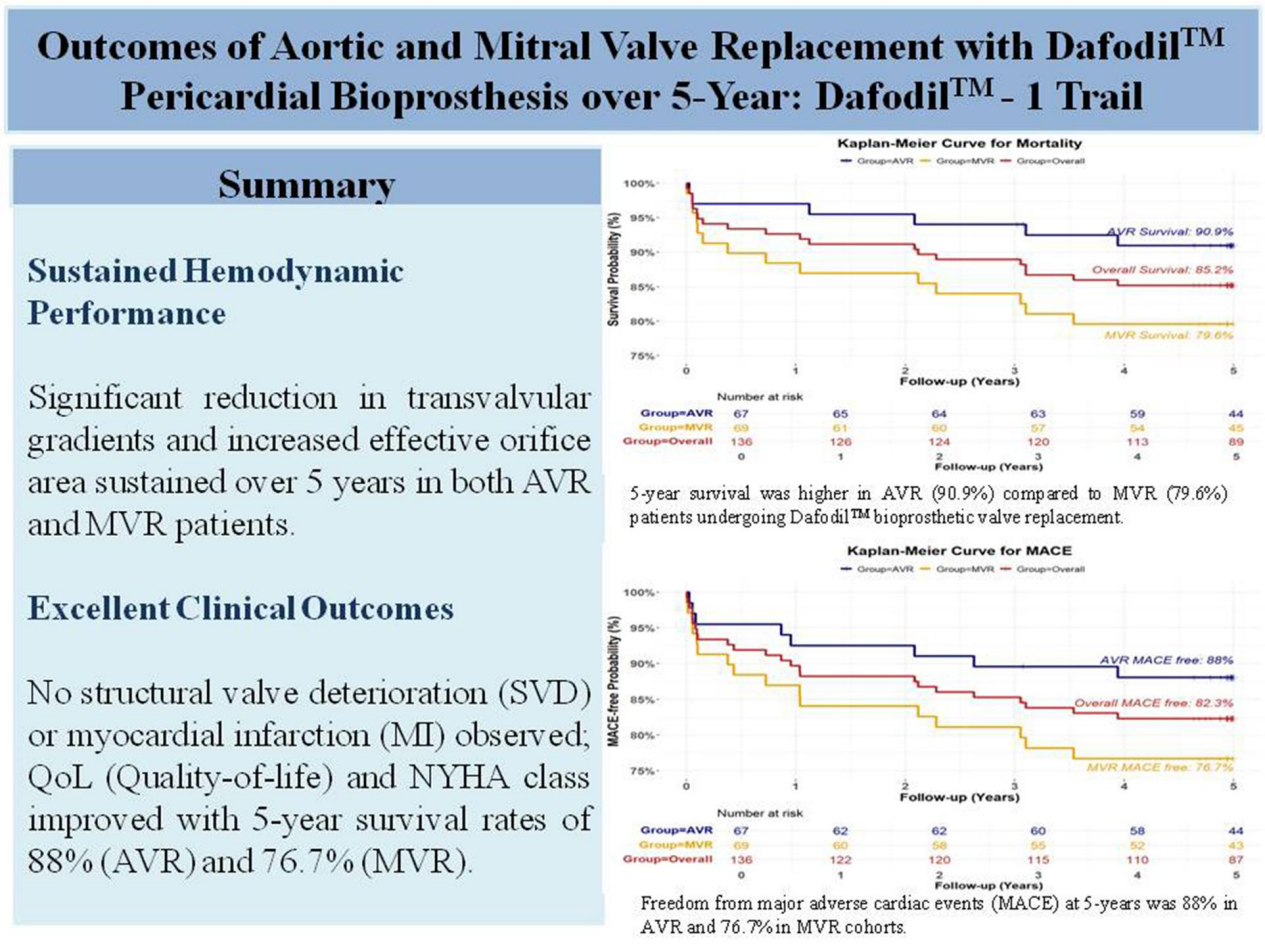
Central illustration: 5-year outcomes of the Dafodil™-1 study.

The 5-year follow-up findings from this study provide encouraging evidence supporting the improved clinical outcomes, safety outcomes, haemodynamic performance, NYHA Functional Class, SF-12 scores of the Dafodil™ pericardial bioprosthesis towards aortic and mitral valve replacement (Figure 4).

### 4.1 Clinical outcomes

A significant finding of this study was that no SVD events were reported in both AVR and MVR group over 5 years. This finding for AVR group is in alignment with four other contemporary bovine surgical aortic prosthesis including Cingular bovine pericardial aortic valve (Cingular Biotech, Shanghai, China) (3), Model 11000A aortic bioprosthesis (Edwards Lifesciences, Irvine, CA, USA)-a Polish study (8), Model 11000A aortic bioprosthesis (Edwards Lifesciences) from COMMENCE Trial (10), and Avalus™ bioprosthesis (Medtronic, Minneapolis, MN, USA) from PERIGON Pivotal Trial (11) wherein they all reported 100% freedom from SVD at 5-year follow-up from a grand total of 1,344 patients. A significant factor contributing to superior durability of Dafodil™ pericardial bio prosthesis is its tri-leaflet design within an elgiloy alloy stent and proprietary AntiCa + anti-calcification treatment that addresses calcification. This anti-calcification property resembles to EIP™ technology in Inspiris Resilia™ aortic valve, AOA™ anti-calcification technology in Avalus™ bioprosthesis.

It is worth noting that the definition of SVD used in this study was primarily based on Akins et al. (2008)'s Guidelines for reporting mortality and morbidity after cardiac valve interventions (15). This definition differs from the Valve Academic Research Consortium-2's, which is utilized in many contemporary transcatheter AVR studies and takes haemodynamic compromise into account. Since some patients may not be able to receive reintervention, our conservative definition may understate SVD. With regards to valve-related complications including major bleeding, prosthetic valve endocarditis, explants and valve thrombosis, Dafodil™ bio prosthesis proved superior to that of Avalus™ bioprosthesis (11).

In our study, MVR group constitutes a younger cohort (average age 49 ± 14.4 years) than that of AVR group. It is reported that young patients who have been implanted with bioprosthetic mitral valves have a higher risk of severe valvulopathy and reoperation, which could be a significant factor in their poorer survival if there are no survival gains from fewer major bleeding events and stroke (16, 17). This might explain reporting of complications [all bleeding (major & minor), explant, thrombosis, prosthetic valve endocarditis, and valve related reoperation] that occurred only in the MVR group, reinforcing the valve's favourable performance in the aortic position.

### 4.2 Safety

Our study recorded excellent safety profile with higher overall survival rates (90.9% and 79.6%) and MACE-free rate (88% and 76.7%) at 5-year follow-up in the AVR group and the MVR group, respectively. Our results for AVR group are comparable with Korean study (18) that reported overall survival rate (87.8%) for 2,381 patients. In fact, our study results for AVR group are better than Carpentier Edwards AVR implantation study (19) that reported 5-year survival rate of 72.1% for 6,096 patients. Our results for MVR group are in agreement with Schnittman et al.'s (20) long-term study on survival and major morbidity after mitral valve replacement in 2,727 patients. Further, it is known that the long-term survival benefit through MVR implantation comes at the cost of a higher risk of major bleeding, stroke or systemic embolism events in all patients, which is evident from Ribeiro et al.'s study (21) that reported two cases of stroke at 5-year follow up. Two patients with stroke and one patient with all bleeding (major & minor) at 5-year follow-up in the current study reflects this observation. One patient implanted with the Dafodil mitral valve demonstrated restricted leaflet mobility at the 5-year follow-up, but without any paravalvular regurgitation or haemodynamic changes. Importantly, there was no evidence of structural valve deterioration (SVD), as the findings did not indicate any intrinsic abnormality of the prosthesis such as wear, fatigue failure, stress fracture, occluder escape or suture line disruption of components, that could lead to stenosis or regurgitation. Therefore, although leaflet mobility was restricted, the absence of intrinsic abnormality and paravalvular regurgitation was suggestive of no SVD.

### 4.3 Haemodynamic performance

Haemodynamic performance is a crucial aspect of the tissue valves. The Dafodil™ valve demonstrated favourable haemodynamic characteristics in this study, with mean transvalvular gradients ranging from 10 to 15 mmHg over 5 years.

The haemodynamic performance of the Dafodil™ valve remained stable and comparable to that of established valves such as Perimount Magna Ease™, Avalus™, and MITRIS RESILIA™ (11, 22, 23). The mitral mean gradient and indexed EOA support sustained leaflet function over time. In contrast to MITRIS RESILIA™, which reported one SVD in 82 patients at 5 years, Dafodil™ demonstrated complete freedom from SVD in both cohorts. At 5-year follow-up, the Dafodil™ AVR group exhibited a mean transvalvular gradient of 11.2 ± 5.2 mmHg and EOA of 1.72 ± 0.5 cm^2^, indicating stable leaflet mobility. These results were on par with the COMMENCE trial (transvalvular gradient: 11.5 ± 6.0 mmHg; EOA: 1.6 ± 0.5 cm^2^) and PERIGON trial (transvalvular gradient: 12.5 ± 4.6 mmHg; EOA: 1.43 ± 0.3 cm^2^). The haemodynamic durability of Dafodil™ at the 5-year follow-up also extended to mitral implants, with MVR patients showing sustained reductions in transvalvular gradients and improvements in indexed EOA (1.28 ± 0.4 cm^2^/m^2^), highlighting the valve's versatility and performance across both anatomical positions.

In comparison to the MITRIS RESILIA™ mitral valve, which reported one SVD event in 82 patients at 5 years (23) and existing predicate valves such as Medtronic's Mosaic™ (freedom from SVD ∼94% at 10 years; mean gradients 4 to 5 mmHg) (24) and Perimount Magna™ (freedom from SVD ∼90% at 5 years; mean gradients 5 to 8 mmHg) (22), the Dafodil™ valve demonstrates comparable or superior mid-term durability and haemodynamics, with no observed valve-related complications at 5 years.

### 4.4 NYHA functional class

The 5-year follow-up trial outcomes also examined statistically significant improvements in NYHA classes for both AVR and MVR group, reporting that 78.4% and 86.36% of AVR and MVR patients were NYHA class I at 5 years, up from 4.5% and 2.9%, respectively at baseline. Our study results for AVR group are in alignment with another 5-year outcome study (25) involving Avalus bioprosthesis (Medtronic) through SAVR.

Also, statistically significant transition in MVR group from baseline for Class I (86.36%) and Class II (13.64%) at 5-year reflect good haemodynamic performance, no SVD and myocardial infarction case post implantation of mitral Dafodil™ bio-prosthetic valve. Our study results for MVR group are consistent with long term durability study (26) involving Carpentier-Edwards PERIMOUNT bioprosthesis (Edwards Lifesciences, Irvine, Calif).

### 4.5 Health-related QoL outcomes

The physical and mental components of the SF-12 showed statistically significant improvement when compared at 5-year follow-up with baseline values, in both AVR and MVR groups.

For MVR group, our results for 5 years follow-up are in line with the 3-year Dafodil™-1 trial (13) SF-12 outcomes. Also, for MVR group, our results are all the more remarkable when one considers that MVR group constitutes a younger cohort (average age 49 ± 14.4 years) and these comparatively young patients after mitral valve replacement surgery need to lead an active lifestyle including professional work attendance and fulfilling social commitments. However, there are no published studies reporting SF-12 scores at 5-year follow-up in severe symptomatic AS patients with bio prosthetic mitral valve replacement.

Irrespective to AVR or MVR group, the short-term need for anticoagulant (most commonly Warfarin) post bio prosthetic valve replacement is generally well tolerated by symptomatic severe AS patients and does not impact quality of life.

## 5 Limitation

The Dafodil™-1 trial, which provides encouraging long-term safety, durability, clinical efficacy and haemodynamic data for up to 5 years, has several limitations. This study employed a non-randomized, single-arm design without an active comparator, which restricts the capacity to assess the superiority or non-inferiority of the Dafodil™ valve to other commercially available bio prostheses and introduces potential bias in outcome interpretation. Also, the lack of blinding and independent adjudication of clinical events may have resulted in assessment bias, particularly in the subjective evaluation of outcomes. Further, site-specific variability in clinical practice and data reporting could have been caused by the lack of procedural standardization across participating centers. Nevertheless, uniform eligibility criteria, imaging protocols, and follow-up timelines were applied to mitigate heterogeneity to some extent. Although the inclusion of additional patients enhanced the study's statistical power and improved the generalizability of its findings, the overall sample size remained relatively limited (*n* = 136), particularly when stratified by valve position (aortic vs. mitral), thereby reducing the robustness of subgroup analyses and potentially limiting the precision of outcome estimates. Nonetheless, no cases of SVD, MI, or valve-related reinterventions were observed over the 3- to 5-year period, the relatively short mid-term follow-up precludes conclusions about long-term valve durability, which is typically assessed over 10–15 years.

## 6 Conclusions

The Dafodil™ bioprosthesis demonstrated sustained haemodynamic performance, favourable clinical outcomes, satisfactory clinical safety, improved QoL, and NYHA functional class over a 5-year follow-up in both the AVR and MVR cohorts. It was interesting to note that there were no cases of SVD or valve - related reintervention in the AVR group, and age was found to be a significant factor in mortality in the MVR cohort. These findings reinforce the role of the Dafodil™ valve as a reliable surgical bioprosthesis and add to the evidence supporting its potential clinical utility pending further long-term validation, particularly in younger and middle-aged patients requiring AVR. These results are especially relevant in resource-limited settings, where device accessibility, affordability, and long-term outcomes are key considerations. The competitive clinical profile of Dafodil™ was similar to those of established bioprosthetic valves like Avalus™, INSPIRIS RESILIA™, MITRIS™, and Perimount Magna Ease™. Nevertheless, extended follow-up beyond 5 years is essential to further validate the valve's long-term safety, durability and clinical efficacy.

## Data Availability

The original contributions presented in the study are included in the article/Supplementary Material, further inquiries can be directed to the corresponding author.
