# Supplementary material for: Outcomes of aortic and mitral valve replacement with Dafodil™ pericardial bioprosthesis over 5-years: Dafodil™-1 trial
Source: Front Cardiovasc Med. 2026 Jan 9;12:1672315. doi: 10.3389/fcvm.2025.1672315 (PMC12828671; doi:10.3389/fcvm.2025.1672315)
Supplement: Supplementary file 1 [file Datasheet1.pdf]

Supplementary Material

Supplementary Table S1: Key study endpoints

Clinical endpoints	Haemodynamic endpoints (Baseline, discharge, 1 year, 3 years, and 5 years)	NYHA functional class (Baseline, discharge, 1 year, 3 years, and 5 years)
All-cause mortality	Peak pressure gradient	Class-I
Cardiovascular death	Mean pressure gradient	Class-II
Myocardial infarction	Effective orifice area	Class-III
Stroke	Effective orifice area index	Class-IV
MACE	Aortic regurgitation grade	
Major & minor bleeding		
Valve thrombosis		
Structural valve deterioration		
Repeat hospitalization		
Conduction disturbances and arrhythmias/ permanent pacemaker implantation		
Prosthetic valve endocarditis		
Major paravalvular leak		
Explant		
Haemolysis		
Valve-related re-operation		

NYHA: New York Heart Association, MACE: Major Adverse Cardiovascular Event

Supplementary Table S2: Characteristics of the subjects at the baseline

Variables	AVR group; n/N = 67/136	MVR group; n/N = 69/136	p-value
Age, years	60.2 ± 8.3	49.7 ± 14.4	<0.001
**Gender, n (%)**			
Male	44 (65.7)	28 (40.6)	0.005
Female	23 (34.3)	41 (59.4)	
Body mass index (kg/m^2)	24.2 ± 4.8	21.6 ± 5.0	0.003
Body surface area (m^2)	1.6 ± 0.2	1.5 ± 0.3	0.015
**Cardiac status, n (%)**			
Stable angina	7 (10.5)	4 (5.8)	0.496
Unstable angina	0	1 (1.5)	1.0
Asymptomatic	60 (89.6)	64 (92.8)	0.722
**Cardiac rhythm, n (%)**	**N=54**	**N=44**	
Sinus rhythm	1 (1.9)	32 (72.7)	<0.001
Atrial fibrillation	52 (96.3)	11 (25.0)	
Atrial flutter	1 (1.9)	1 (2.3)	
**Medical history, %**			
Diabetes mellitus	13.4	1.5	0.008
Hypertension	28.4	10.1	0.013
Myocardial infarction	6.0	0	0.056
Family history of CAD	6.0	0	0.056
Cerebrovascular events	3.0	2.9	1.0
Congestive heart failure	3.0	1.5	0.616
Heart rate, bpm, (mean ± SD)	77.1 ± 13.0	79.5 ± 13.4	0.975
Diastolic blood pressure, mmHg (mean ± SD)	73.4 ± 9.1	76.3 ± 13.9	0.058
Systolic blood pressure, mmHg (mean ± SD)	123.4 ± 16.6	120.8 ± 15.6	0.725
**Haemodynamic variables, mean ± SD**			
Peak Pressure gradient (mmHg)	80.4 ± 4.0	16.6 ± 8.3	NA
Mean pressure gradient (mmHg)	51.2 ± 24.1	8.8 ± 5.0	NA
EOA (cm^2)	0.9 ± 0.6	1.1 ± 0.6	NA

Variables	AVR group; n/N = 67/136	MVR group; n/N = 69/136	p-value
iEOA (cm^2/m^2)	0.5 ± 0.3	0.7 ± 0.4	NA
LVEF (%)	54.8 ± 12.8	NA	NA
**NYHA functional class, n (%)**			
Class-I	3 (4.5)	2 (2.9)	0.045
Class-II	10 (14.9)	5 (7.2)	
Class-III	54 (80.6)	59 (85.5)	
Class-IV	0	3 (4.3)	

AVR, aortic valve replacement; CAD, coronary artery disease; MVR, mitral valve replacement; EOA, effective orifice area; iEOA, indexed EOA; LVEF, left ventricular ejection fraction; NYHA, New York Heart Association. Data are presented as mean ± SD or n (%).

Supplementary Table S3: Comparison between late outcomes and early outcomes towards quality of life (SF-12 Score) assessment in patients implanted with aortic Dafodil™ bio-prosthetic valve

SF-12 Assessment	Early Outcomes		Late Outcomes			
	**Baseline (n=67)**	**One-Month Follow up (n=65)**	**One-Year Follow up (n=63)**	**Four-Years Follow up (n=53)**	**Five-Years Follow up (n=53)**	**p-value (Baseline vs 5 Year)**
**Aortic Valve Group, mean ± SD**						
PCS	33.82 ± 7.41	37.08 ± 5.53	45.21 ± 7.37	47.10 ± 7.96	46.38 ± 7.44	<0.0001
MCS	43.43 ± 9.64	47.47 ± 8.55	53.02 ± 7.35	52.69 ± 8.70	53.06 ± 9.2	<0.0001
PCS+MCS	77.25 ± 19.91	84.55 ± 10.98	98.23 ± 11.07	99.79 ± 13.1	99.45 ±13.41	<0.0001
**Mitral Valve Group, mean ± SD**						
	**(n=69)**	**(n=62)**	**(n=60)**	**(n=47)**	**(n=44)**	
PCS	32.05 ± 6.36	39.70 ± 6.54	43.42 ± 8.58	47.00 ± 6.67	44.78 ± 7.91	<0.0001
MCS	43.49 ± 10.15	45.15 ± 8.34	52.24 ± 6.79	52.2 ± 6.04	51.82 ± 8.63	<0.0001
PCS+MCS	75.53 ± 12.11	84.85 ± 10.25	95.66 ± 10.82	99.2 ± 10.64	96.6 ± 11.35	<0.0001

Physical Component Score (PCS) Component Score (MCS)

Supplementary Table S4: Haemodynamic data of patients with Small Aortic Annulus (n=55).

Echo parameter	Baseline	Discharge	1-month	6-month	1-year	2-year	3-year	4-Year	5-year	P-value
**19 mm group**										
Peak Pressure Gradient (mmHg)	85.9±35.9	29.0±12.3	25.8±7.1	25.1±7.2	28.4±10.3	27.6±10.4	24.3±13.7	23.37 ± 8.9	23.55 ± 8.55	<0.001
Mean Pressure Gradient (mmHg)	54.3±25.1	15.4±6.7	13.8±4.3	13.8±4.3	16.3±7.1	14.7±5.3	12.5±6.3	11.42 ± 4.15	12.1 ± 4.75	<0.001
EOA (cm^2)	0.8±0.3	1.5±0.5	1.4±0.4	1.6±0.4	1.5±0.4	1.4±0.3	1.6±0.5	1.59 ± 0.46	1.46 ± 0.36	<0.001
EOAI (cm^2/m^2)	0.5±0.2	0.9±0.3	0.9±0.2	1.0±0.3	1.0±0.3	0.9±0.2	1.0±0.3	0.98 ± 0.35	0.88 ± 0.3	0.004
**21 mm group**										
Peak Pressure Gradient (mmHg)	87.7±26.0	27.1±17.0	22.5±4.9	22.2±6.4	22.8±8.2	20.1±6.7	20.5±12.3	20.47 ± 8.89	21.6 ± 10.96	<0.001
Mean Pressure Gradient (mmHg)	56.4±19.7	14.9±9.0	12.0±2.8	12.1±4.4	12.8±5.4	10.7±4.4	10.7±6.5	10.96 ± 5.81	11.82 ± 6.4	<0.001
EOA (cm^2)	0.8±0.3	1.6±0.6	1.7±0.3	1.7±0.4	1.7±0.4	1.8±0.4	1.9±0.3	1.48 ± 0.37	1.72 ± 0.46	<0.001
EOAI (cm^2/m^2)	0.5±0.2	1.0±0.4	1.0±0.2	1.0±0.3	1.0±0.2	1.0±0.2	1.1±0.4	0.97 ± 0.33	1.05 ± 0.41	<0.001

EOA, effective orifice area; EOAI, indexed EOA.
